# Are Autonomous and Controlled Motivations School-Subjects-Specific?

**DOI:** 10.1371/journal.pone.0134660

**Published:** 2015-08-06

**Authors:** Julien Chanal, Frédéric Guay

**Affiliations:** 1 Department of Psychology, Methodology and Data Analysis Group, Faculty of Psychology and Educational Sciences, University of Geneva, Geneva, Switzerland; 2 Distance Learning University, Brig, Switzerland; 3 Faculty of Educational Sciences, Laval University, Quebec, Canada; University of Rome, ITALY

## Abstract

This research sought to test whether autonomous and controlled motivations are specific to school subjects or more general to the school context. In two cross-sectional studies, 252 elementary school children (43.7% male; mean age = 10.7 years, SD = 1.3 years) and 334 junior high school children (49.7% male, mean age = 14.07 years, SD = 1.01 years) were administered a questionnaire assessing their motivation for various school subjects. Results based on structural equation modeling using the correlated trait-correlated method minus one model (CTCM-1) showed that autonomous and controlled motivations assessed at the school subject level are not equally school-subject-specific. We found larger specificity effects for autonomous (intrinsic and identified) than for controlled (introjected and external) motivation. In both studies, results of factor loadings and the correlations with self-concept and achievement demonstrated that more evidence of specificity was obtained for autonomous regulations than for controlled ones. These findings suggest a new understanding of the hierarchical and multidimensional academic structure of autonomous and controlled motivations and of the mechanisms involved in the development of types of regulations for school subjects.

## Introduction

Self-determination theory (SDT) endorses an organismic viewpoint, whereby students are inherently proactive and have capabilities that will flourish when they are provided with the necessary nutriments at school [1]. SDT describes two categories of regulation that can drive behaviors: autonomous (i.e., behaviors are performed out of interest and enjoyment or for their inherent value) and controlled (i.e., behaviors are performed under internal or external pressure). Numerous studies have shown that when students are motivated by autonomous rather than controlled motivation, they experience more positive outcomes [2,3, 4]. Although these two regulation categories have been assessed in various school subjects (i.e., mathematics, first languages, second languages, and physical education) or in school in general, only a few studies have investigated them simultaneously for various school subjects and at various levels of generality.

**Fig 1:**
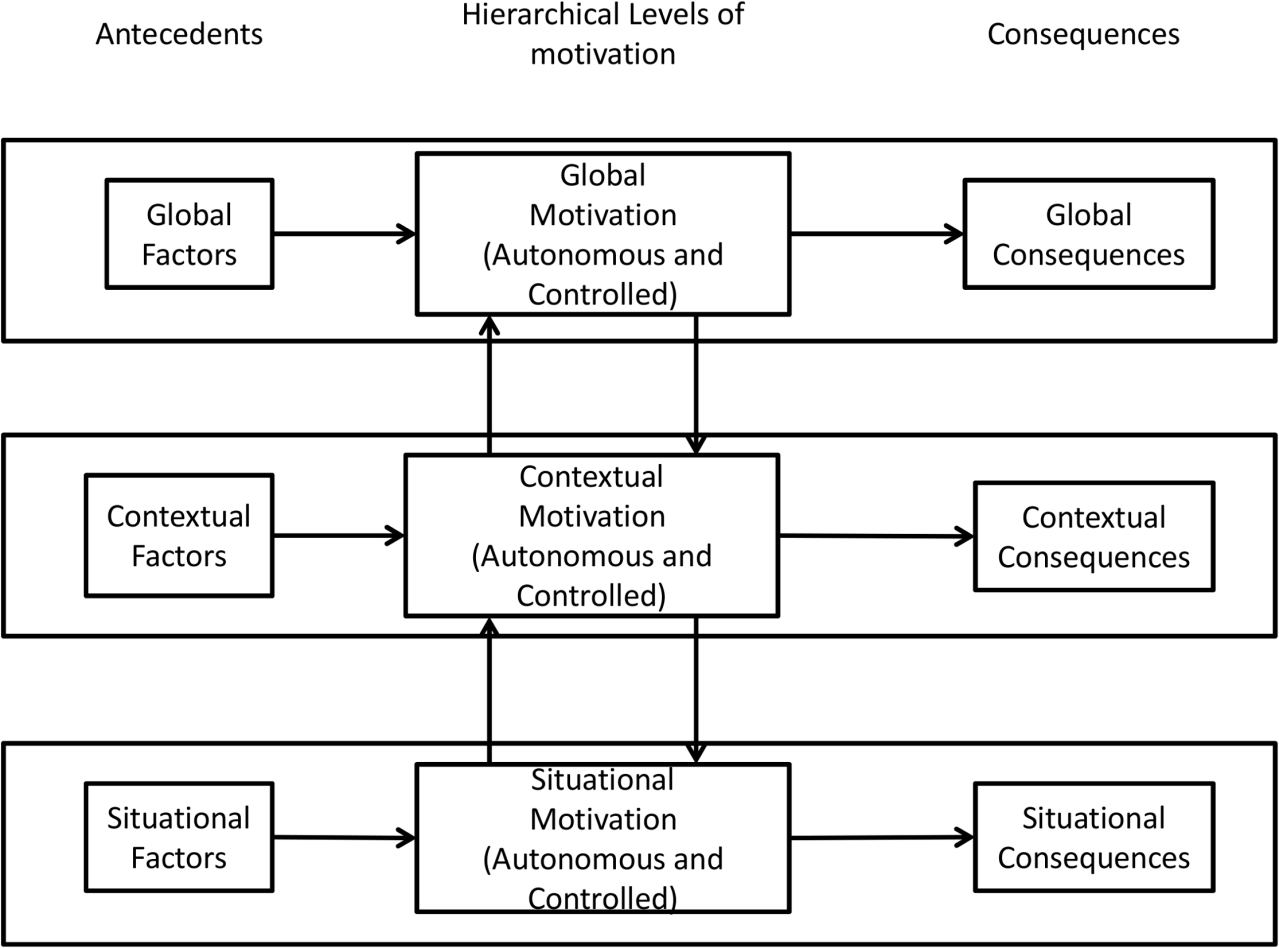
Vallerand’s (1997) hierarchical model of intrinsic and extrinsic motivation.

The two present studies were designed to empirically test whether autonomous and controlled motivations are school-subject-specific. Indeed, the Hierarchical Model of Intrinsic and Extrinsic Motivation (HMIEM; Fig 1) [5] posits specific effects at a given level connecting motivation to its antecedents and consequences (horizontal arrows). However, no studies to our knowledge have focused on testing specifically whether school subject measures were equally school-subject-specific in autonomous and controlled motivations.

This is an important research avenue, because the examination of relationships between academic or school-subject self-perceptions (such as self-concept or motivation) or/and other constructs (i.e., academic achievement) requires an appropriate structural model. In both studies, we considered autonomous and controlled motivations at an academic level (i.e., in school in general) and at four school-subject-specific levels (i.e., mathematics, science, writing, and reading in Study 1 or mathematics, French, English and physical education in Study 2). Below, we review studies that investigated the links between autonomous and controlled motivations for a given school subject and the relationships between these regulation types across various school subjects. Finally, we present the results from studies that focused on the hierarchical aspect of these two motivation categories [5].

### Autonomous and controlled motivation for a given school subject

SDT postulates that there are two main categories of motivations that underlie students’ behaviors, namely intrinsic and extrinsic [1]. Intrinsic motivation occurs when students are motivated by the inherent pleasure they feel when performing an activity. In contrast, extrinsic motivation occurs when students are motivated by the external consequences of performing a given activity. SDT proposes various types of extrinsic motivation characterized by various degrees of self-determination (i.e., the degree to which the regulation is integrated into the self). From high to low level of self-determination, these types are identified regulation, introjected regulation, and external regulation. Identified regulation implies that individuals perform a behavior because of the inherent value they attach to it; they do so by choice or because they consider it important. This type is followed by introjected regulation, in which behaviors are regulated to avoid guilt or shame, to act self-protectively or to present a positive image to others. Finally, external regulation refers to behaviors performed under external sources of pressure such as rewards, punishments, or constraints.

To predict ouctomes, researchers have used either each regulation type separately [2], or a relative autonomy index in which the scores for each type of regulation are algebraically combined into a single composite score [6]. Alternatively, they have used only two broad categories of motivation, namely autonomous and controlled [7]. Autonomous motivation encompasses intrinsic and identified regulations, whereas controlled motivation includes introjected and external regulations. Previous SDT studies have always considered regulation types as equally specific to the school subject investigated sometimes using them separately and sometimes combined.

To present our literature review more efficiently, we refer to autonomous and controlled motivations. However, we examined each regulation type separately to test our hypotheses.

### The multidimensional aspect of autonomous and controlled motivation: Between-school-subject differentiation

Traditionally, motivation researchers have assessed variations in motivation across various school subjects [8, 9, 10]. Support has been obtained for *between-school-subject* differentiation effects with respect to intrinsic motivation. Intrinsic motivation for various school subjects (reading, mathematics, social studies, and science) were more strongly associated with other motivational constructs within the school subject concerned than with those for other school subjects [11, 12].

In this study, we wanted to replicate the between-school-subject differentiation effect obtained for intrinsic motivation and to extend our analysis to identified, introjected, and external regulations. We postulated that differentiation effects would be obtained for these four types of motivation, but that the intensity of this differentiation would differ across types. We therefore expected between-school-subject differentiation to be stronger at the higher end of the self-determination continuum (intrinsic motivation) and lower as self-determination declines. Since intrinsic motivation is not instrumentally focused, it should be more differentiated than extrinsic motivation. More specifically, students perform various activities offered at school so they can discover which ones they enjoy and which ones they do not. Identified regulation should be less differentiated than intrinsic motivation, because this regulatory process is less tied to the inherent characteristics of the activity and more governed by the endorsement of cultural values [13]. In fact, students may understand that reading, writing, science and mathematics are important for their development as individuals, even though they may identify with one subject more than another (they value one more than another). Because introjected regulation implies dealing with an internal impetus and cognitive-affective consequences that are operative across various activities [13], introjected regulation should be less differentiated than identified regulation. Finally, we posited that external regulation would not be very much differentiated among school subjects, because it involves managing an external impetus that may be operative across school subjects. For example, an elementary school teacher who uses external contingencies to motivate children will do so not only in mathematics, but in other subjects as well. Moreover, the fact that high school entails more extrinsic controls and rigid constraints could explain why most high school students would develop undifferentiated types of external regulation across school subjects. In fact, external regulation, while not always adaptive [1, 3], can inevitably develop in many school subjects in order for students to meet environmental demands.

To the best of our knowledge, only Guay et al. [14] have extended such a between-school-subject differentiation analysis to other types of autonomous and controlled motivations. These authors’ [14] results demonstrate that the size of the correlations connecting between-school-subject regulations differed depending on the level of self-determination. More specifically, for three school subjects (mathematics, reading, and writing), the correlations among intrinsic motivations were lower than those among identified motivations. Moreover, for these school subjects, the correlations among identified regulations were lower than those among controlled motivations (introjected and external regulation were considered jointly). Finally, the results indicated that these differentiation effects were more pronounced for older children. These findings support the idea that between-school-subject differentiation exists for autonomous and controlled motivations but that this differentiation effect is more pronounced for autonomous motivation than for controlled motivation.

### The hierarchical aspect of autonomous and controlled motivation: Between-level differentiation

The HMIEM (see Fig 1) assumes that motivation is differentiated across levels of generality, which means that autonomous and controlled motivations differ according to contextual and situational levels. This conceptual framework was designed primarily to organize and understand the core mechanisms underlying the relationships between the determinants and consequences of motivation at various levels of generality. Numerous studies have demonstrated the specificity of the antecedents and consequences of motivation at various levels [15], and others have shown bottom-up and top-down effects between levels [16, 17]. However, to our knowledge, no studies have investigated a model in which contextual (i.e., school in general) and various situational (i.e., school subject) motivations are assessed simultaneously. Only Ntoumanis and Blaymires [18] have tested the specificity effect between levels. They showed that self-determined, or autonomous, contextual motivation (conceptualized through a relative autonomy index) for physical education was related more strongly to self-determined motivation for a particular sport practiced in physical education than to self-determined motivation in science. However, compared to motivation in a particular sport, motivation for science was related more strongly to contextual motivation in school.

As stated above, the HMIEM model postulates autonomous and controlled motivations as hierarchically specific structured constructs. However, when modeling between-level motivation relationships, studies based on the HMIEM model have consistently used the relative autonomy index, which employs an algebraic formula to determine whether individuals are more motivated by autonomous than by controlled motivation [16]. Given that autonomous motivation has been shown to be more differentiated across school subjects, whereas controlled motivation has not [14], this aggregation of regulation types could be misleading because we cannot determine whether each regulation is differentiated across various school subjects and between levels. More specifically, the between-level differentiation effect could differ depending on the regulation type, which would explain why some regulations are more differentiated across school subjects than others [14]. A larger between-school-subject differentiation may be the result of a larger between-level differentiation effect and, alternatively, a lower between-school-subject differentiation may be the result of a lower between-level differentiation effect. In other words, we believe that the degree of specificity of the autonomous motivation and the controlled motivation would differ at the situational level. As autonomous motivation has been found to be **more differentiated** than controlled motivation between school subjects [14], we postulate that autonomous motivation would be **more school-subject-specific** than controlled motivation.

### Research aims and hypotheses

The main goal of the two studies was to test whether the more autonomous the regulation, the more school-subject-specific it would be, and whether the more controlled the regulation, the less school-subject-specific it would be. Intrinsic and identified regulations (i.e. autonomous) were postulated to be more school-subject-specific than introjected and external regulations (i.e., controlled). More specifically, we posited that autonomous motivation constructs at the situational level would explain more variance of the items assessed at the situational level than controlled motivation. As autonomous motivation is hypothesized to be more specific, we also posited that we will find more relationships between autonomous motivation and specific constructs associated with the situational level (e.g., students’ self-concept and achievement) than for controlled motivation. More specifically, we postulated autonomous motivation to be more related than controlled motivation to students’ self-concept and achievement, and that these relationships would be stronger in corresponding school subjects than in non-corresponding ones, reflecting the school-subject-specificity of the measure. We used academic self-concept and achievement because Marsh et al. have shown in numerous studies that these variables are differentiated across school subjects [19] and because these constructs are correlated with motivation types [20]. The theoretical implications of this hypothesis are substantial. More specifically, until now most researchers have considered autonomous and controlled motivations in a given school subject as equally specific [13]. We believe that the between-level differentiation effect of autonomous and controlled motivations is so conceptually central that it may be used to refine not only our understanding of the relationships between important constructs involved in students’ achievement in school, but also our comprehension of the determinants of students’ levels of autonomous and controlled motivations in specific learning situations.

To test our assumptions, two studies were conducted among elementary and secondary school children to determine the school-subjects-specificity of the regulation types.

## Method

### Samples

#### Study 1

The participants were 252 French-speaking Grade 5 students (43.7% male; mean age = 10.7 years; SD = 1.3 years) attending six public elementary schools in the Canton of Geneva, Switzerland. These students had participated in a study initiated in 2009. The data presented in this article were obtained from the 2011 data collection, which was designed to test the hypotheses proposed in this manuscript.

#### Study 2

The participants were 334 French-speaking students (113 Grade 7, 101 Grade 8 and 120 Grade 9; 49.7% male; mean age = 14.07 years; SD = 1.01 years) from the same public junior high-school in the Canton of Geneva, Switzerland.

### Procedure

Questionnaires were administered in the classroom by an experienced research assistant. The following instructions were given to all children: “This is a chance to help me find out how you feel. It is not a test. There are no right or wrong answers, and everyone will have different answers. I will ask you to read each question and then ask you to write how you feel about it by circling a number on the scale ranging from 1 (*Totally disagree*) to 5 (*Totally agree*). Make sure your answers show how you feel about yourself. We will not show your answers to anyone else. If you do not understand a sentence or a word in a sentence, please tell me.”

The same procedure was used in both studies except that the Likert-type scale ranged from 1 (*Totally disagree*) to 7 (*Totally agree*) in Study 2.

These studies were approved by the "Commission de recherche dans les écoles" of canton of Geneva. No written consent was required from the children because the data was obtained and analyzed anonymously. This procedure was approved by the Commission, and the department of public instruction obtained the consent of school principals who wanted to participate in the study. Once the school principals had agreed to participate, teachers were informed that our study would take place in their classes. The student's participation in the survey was voluntary and they had the option to stop participating or to withdraw if they wished.

### Measures

#### Study 1. Autonomous and controlled regulations

The Elementary School Motivation Scale (*E* ***SMS*)** [14] was adapted and administered to the children to assess their regulation types for four school subjects (mathematics, science, writing, and reading). A contextual version of the ESMS was also administered to the children.

The original version of the ESMS contains 27 items measuring intrinsic (“I enjoy learning to read”, “Learning to read interests me a lot”, and “I read even when I don’t have to”), identified (“I can learn many useful things by learning to read”, “I learn to read to learn many things”, and “In life, it’s important to learn how to read”), and controlled regulations for three school subjects (mathematics, writing, and reading). Introjected and external regulations were assessed jointly under the concept of controlled motivation because the ESMS was designed to assess motivation in a sample of young children (i.e., 6–8 years old). In this study, we decided to measure introjected (“I learn to read because I would feel bad if I didn’t”, “I feel pressured to learn to read”, and “I read to show others how good I am”) and external (“I learn to read to get a nice reward”, “I learn to read to please my parents or my teacher”, and “I learn to read to avoid problems with my teacher or my parents”) regulations separately and to add a fourth school subject and a contextual measure. The questionnaire used therefore contained 60 items (i.e., three items for four regulations in five dimensions: specific school subjects plus the school level). The same three items were used to assess each regulation at the school subject and contextual levels. For example, the same intrinsic motivation item was used for all school subjects and for the contextual measure. The children were asked to rate how much they agreed with each item on a five-point scale from 1 (*Totally disagree*) to 5 (*Totally agree*).

#### Students’ self-concept

Three items selected from the Academic Self-Description Questionnaire [21, 22] were assessed for the four school subjects and the contextual level, for example, “I have always done well in reading (writing, mathematics, science, school)”, “Reading (writing, mathematics, science, school) is easy for me”, and “I learn things quickly in reading (writing, mathematics, science, school)”.

#### Teachers’ ratings of academic achievement in each school subject

Teachers were asked to provide us with the students’ achievement in each of the four school subjects as well as a global appreciation of their achievement at school. They were asked to assess each student’s performance on a five-point scale ranging from 1 “Among the worst students of the class” to 5 “Among the best students of the class”.

#### Study 2. Autonomous and controlled regulations

An adapted version of the Academic Motivation Scale (*AMS*) [23] was administered to the children.

The same three items were used to assess each regulation at the school subject and contextual levels (mathematics, French, English and physical education). For example, the same intrinsic motivation item was used for all school subjects and for the contextual measure. The children were asked to rate how much they agreed with each item on a seven-point scale from 1 (*Totally disagree*) to 7 (*Totally agree*).

#### Students’ self-concept

Six items were selected from the Academic Self-Description Questionnaire [21, 22] to assess self-concept in the four school subjects and at the contextual level (e.g., “I have always done well in French (mathematics, English, physical education, school)”, “French (mathematics, English, physical education, school) is easy for me”, and “I learn things quickly in French (mathematics, English, physical education, school)”.

### Statistical analyses

#### Correlated trait-correlated method minus one model

The above-mentioned hierarchical and multidimensional aspects require statistical models to be built according to hierarchically structured constructs [24]. Among the multiple approaches proposed, the correlated trait-correlated method minus one (CTCM-1) model [25] appeared to be the most suitable model for our research purpose. This method is used in multitrait-multimethod studies to distinguish variances attributable to traits and methods. This modeling procedure has the advantage of combining and disentangling variances in measures attributable to a global (i.e., contextual) trait or to a state or method (i.e., specific) measure. As highlighted by Brunner, Keller, Dieredonck, Reichert, Ugen, Fishbach and Martin [26] regarding academic self-concepts, this model is suitable for hierarchical and multidimensional constructs “because the model is able to account for (a) the subject-specific nature of constructs, (b) the separation of subject-specific (constructs) across domains, and (c) the hierarchical organization of (constructs), with (d) general academic (construct) at the apex” (p.968).

**Fig 2:**
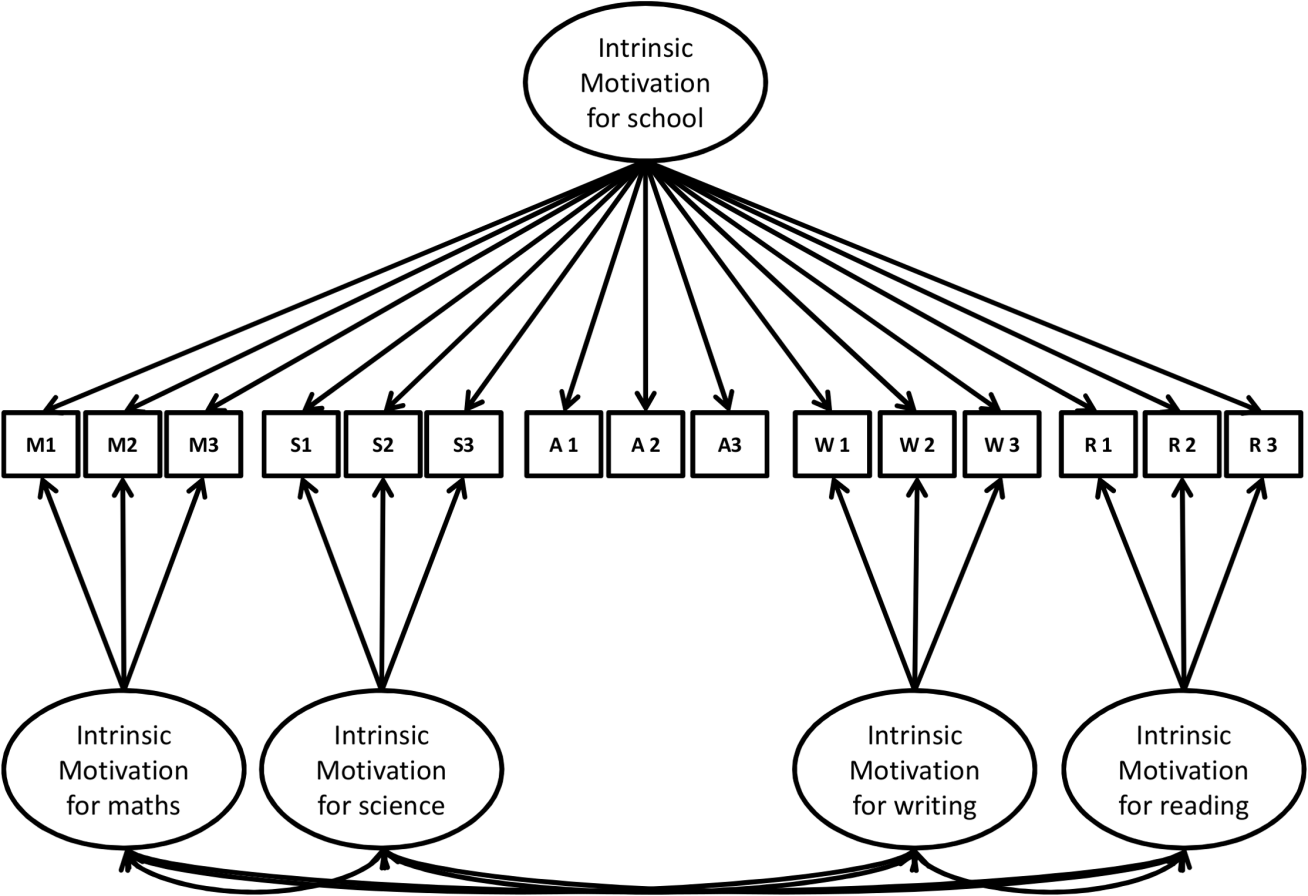
Correlated trait-correlated method minus one model for intrinsic motivation. M1-M3 = items for Mathematics, S1-S3 = items for Science, A1-A3 = items for Academic, W1-W3 = items for Writing, R1-R3 = items for Reading.

This model therefore allows testing of the hierarchical structure of autonomous and controlled academic motivations while taking into account multiple school subjects according to the HMIEM principles postulated by Vallerand [3]. More specifically, the CTCM-1 disentangles the variance in autonomous and controlled motivations attributable to contextual (school) or school subject (e.g., mathematics, science, writing, and reading) levels. Fig 2 presents an example of variance partitioning for intrinsic motivation. More specifically, intrinsic motivation at the school level is considered as a single trait, whereas intrinsic motivations for various school subjects are considered as correlated methods or school subject deviations from the global trait. Intrinsic motivation indicators for the four school subjects are therefore caused not only by specific latent constructs but also by a latent construct for contextual intrinsic motivation. Consequently, the latent factor of the single trait for the four regulations (i.e., intrinsic, identified, introjected and external) at the contextual level comprises the common variance of the school motivation measures that also explained the variance in the school subject indicators. The specific latent factors for each school subject therefore represent deviations from the global trait by capturing the common but specific variance in school subject indicators that is above the common variance at the contextual level. Thus, for indicators assessing each regulation at the contextual level, the method factor is missing, representing the method minus one part of the CTCM-1 model. Crucially, the inclusion of indicators assessing contextual academic motivation combined with the assumption of a missing “method factor” allows the model to be identified and a unique solution to be obtained for all model parameters.

For regulations that are proposed to be school-subject-specific (i.e., autonomous motivation), the common variance captured by the single trait at the higher level would be lower than for regulations that are expected to be less school-subject-specific (i.e., controlled motivation). Consequently, factor loadings associated with the single trait are hypothesized to be weaker for autonomous motivation than for controlled motivation in the upper part of the model (Fig 2). In addition, factor loadings associated with specific deviations from the trait for the four school subjects are hypothesized to be stronger for autonomous than for controlled motivation in the lower part of the model (Fig 2). This should demonstrate that the common variance of indicators is captured more at the specific level for autonomous motivation. Lower variance explained in the lower part of the model would indicate that the single trait has captured the majority of the variance expressed in specific items, and thus the non-specificity to school subjects of the regulation.

#### Missing data

Less than 1% of the data were missing in both studies. Despite this low percentage, it would be highly inappropriate to disregard missing values by using listwise deletion of cases [27]. We therefore performed a full information maximum likelihood (FIML) estimation using Mplus (version 7).

#### Estimation and goodness of fit

All models were tested with maximum likelihood estimation using robust standard errors (MLR estimation). To ascertain model fit, we used the comparative fit index (CFI), the Tucker-Lewis index (TLI), the root mean square error of approximation (RMSEA), the standardized root mean square residual (SRMR), and the chi-square/degrees of freedom ratio (χ2/df). The CFI and TLI vary along a continuum from 0 to 1 where values greater than 0.90 and 0.95 are typically deemed acceptable and excellent fit to the data, respectively. According to Browne and Cudeck [28], RMSEA values less than .05 are considered a good fit, values between .05 and .08 an adequate fit, and values between .08 and .10 a mediocre fit, while values > .10 are unacceptable. A value of less than 0.08 for the SRMR is considered a good fit [29].The chi-square/degrees of freedom ratio (χ^2^/df) is a function of model misfit (χ^2^) compared to model parsimony, as indicated by the degrees of freedom (df) of the model. Smaller χ^2^/df ratios occur when model misfit is lower than model parsimony. In general, a χ^2^/df ratio of less than 2 indicates a relatively good model fit [30].

#### Parallel items

**Fig 3:**
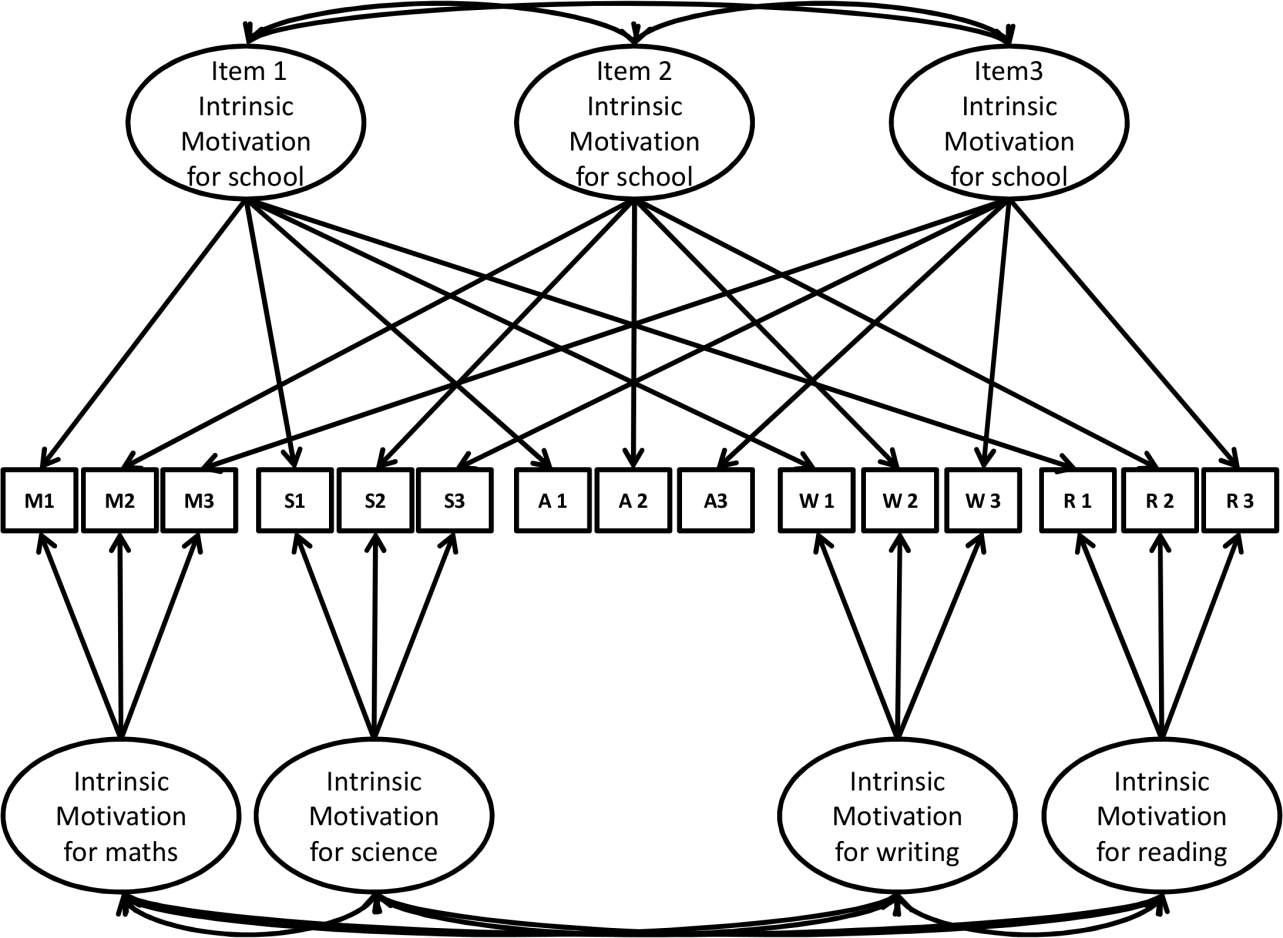
Correlated item-specific trait-correlated method minus one model for intrinsic motivation. M1-M3 = items for Mathematics, S1-S3 = items for Science, A1-A3 = items for Academic, W1-W3 = items for Writing, R1-R3 = items for Reading.

Identical items were used to assess the same regulations across school subjects. We thus created an item-specific latent factor for the same item used to assess a given motivation type for various school subjects and for the contextual measure (see the example for intrinsic motivation in Fig 3).

## Results

Before testing our hypotheses, we began by assessing multiple models in each study. Three models were built for Study 1. The first model included only autonomous and controlled motivation at the school-subject-specific and contextual levels. The four specific school subjects were chosen (1) according to the school programs and (2) in order to calculate correlations between school subjects that are assumed to be more “proximal” on the academic track, such as reading and writing (e.g., a more verbal domain) and mathematics and science (e.g., a more mathematical domain) [31]. The second and third models included self-concept and achievement measures, at the specific and contextual levels. Two models were built in Study 2. As in Study 1, the first model included only autonomous and controlled motivations at the school-subject-specific and contextual levels. The four specific school subjects were chosen according to the school programs. Four school subjects that were compulsory during all the high school years were selected (French, English, mathematics and physical education). The second model included self-concept measures, at the specific and contextual levels. However, we were unable to obtain an assessment of students’ achievement in Study 2.

Table 1 presents the descriptive statistics and reliabilities of the scores for autonomous and controlled motivations as well as for academic self-concepts from the two studies. Table 2 presents the fit indices, which show a good fit to the data for the three models tested in Study 1 and the two models tested in Study 2. In Models 1a and 1b, we estimated the variance components of the latent constructs as well as the relationships among the latent constructs at the school subject level for Study 1 and Study 2, respectively. The variance components and correlations are reported in Tables 3, 4 and 5. Models 2a and 2b included students’ self-concepts for Study 1 and Study 2, respectively. Model 3 included students’ teacher-rated for Study 1 only. In Tables 5, 6 and 7, the correlations between self-concept or achievement and motivational constructs are analyzed to explore the specificity of the motivational dimensions.

**Table 1:** Descriptive statistics and reliabilities for scale scores.

Study 1				Study 2			
Scale score	Mean	SD	Alpha	Scale score	Mean	SD	Alpha
SC-A	3.70	.92	.87	SC-A	4.88	1.31	.94
Int-A	3.83	.94	.79	Int-A	3.59	1.37	.85
Ident-A	4.73	.53	.76	Ident-A	5.63	1.16	.81
Intro-A	2.68	1.03	.60	Intro-A	3.43	1.31	.56
Ext-A	2.43	1.17	.68	Ext-A	2.73	1.58	.71
SC-M	3.82	1.09	.94	SC-M	4.34	1.61	.95
Int-M	3.84	1.06	.81	Int-M	3.28	1.71	.90
Ident-M	4.56	.65	.80	Ident-M	4.99	1.70	.91
Intro-M	2.48	1.09	.66	Intro-M	3.50	1.50	.65
Ext-M	2.21	1.19	.77	Ext-M	2.46	1.51	.73
SC-S	3.94	.95	.91	SC-F	4.92	1.36	.94
Int-S	3.63	1.12	.82	Int-F	3.46	1.57	.89
Ident-S	4.27	.94	.87	Ident-F	5.18	1.42	.85
Intro-S	2.36	1.15	.72	Intro-F	3.23	1.46	.63
Ext-S	2.21	1.24	.79	Ext-F	2.40	1.51	.74
SC-W	3.91	1.12	.88	SC-E	5.56	1.36	.95
Int-W	3.80	1.12	.80	Int-E	4.80	1.74	.92
Ident-W	4.34	.86	.78	Ident-E	5.88	1.35	.88
Intro-W	2.55	1.11	.65	Intro-E	3.50	1.65	.71
Ext-W	2.23	1.21	.78	Ext-E	2.30	1.52	.73
SC-R	4.22	.93	.89	SC-PE	5.48	1.55	.96
Int-R	4.16	1.06	.85	Int-PE	5.05	1.84	.92
Ident-R	4.55	0.77	.84	Ident-PE	4.61	1.63	.81
Intro-R	2.47	1.14	.69	Intro-PE	3.17	1.70	.70
Ext-R	2.23	1.25	.80	Ext-PE	2.21	1.51	.76

*Note*. Scale scores were calculated as the mean of respective item scores. Int = intrinsic motivation; Ident = identified regulation; Intro = introjected regulation; Ext = external regulation; SC = self-concept; A = School; M = mathematics; S = Science; W = writing; R = reading; F = French; E = English; PE = physical education.

**Table 2:** Data fit of structural models.

		χ^2^	χ^2^ /df	CFI	TLI	RMSEA	SRMR
Study 1	Model 1	2293.003	1.55	.917	.901	.047 [.043; .050]	.066
	Model 2	3558.311	1.52	.912	.895	.045 [.042; .048]	.062
	Model 3	2578.224	1.54	.920	.900	.046 [.043; .050]	.064
Study 2	Model 1	2088.225	1.41	.946	.935	.035 [.032; .039]	.055
	Model 2	5060.890	1.43	.934	.925	.036 [.034; .038]	.056

**Table 3:** Reliabilities, consistency and method-specificity coefficients in Study 1.

	Reliability					Consistency				Method-specificity			
	Academic	Math	Science	Writing	Reading	Math	Science	Writing	Reading	Math	Science	Writing	Reading
Intrinsic													
1st Item	.66	.77	.89	.76	.78	.31	.19	.31	.29	.69	.81	.69	.71
2nd Item	.85	.93	.86	.73	.72	.35	.29	.30	.30	.65	.71	.70	.70
3rd Item	.46	.52	.40	.42	.59	.77	.47	.42	.31	.23	.53	.58	.69
**Mean**						**.48**	**.32**	**.34**	**.30**	**.52**	**.68**	**.66**	**.70**
												**Mean**	**.64**
Identified													
1st Item	.64	.65	.76	.56	.72	.42	.15	.50	.44	.58	.85	.50	.56
2nd Item	.51	.74	.75	.77	.77	.52	.33	.46	.53	.48	.67	.54	.47
3rd Item	.74	.59	.68	.59	.67	.48	.19	.51	.90	.52	.81	.49	.10
**Mean**						**.47**	**.22**	**.49**	**.62**	**.53**	**.78**	**.51**	**.38**
												**Mean**	**.55**
Introjected													
1st Item	.72	.68	.68	.73	.74	.95	.76	.83	.72	.05	.24	.17	.28
2nd Item	.76	.90	.82	.70	.80	.80	.68	.88	.92	.20	.32	.12	.08
3rd Item	.59	.78	.72	.87	.74	.95	.75	.76	.82	.05	.25	.24	.26
**Mean**						**.90**	**.73**	**.82**	**.82**	**.10**	**.27**	**.18**	**.18**
												**Mean**	**.18**
External													
1st Item	.69	.83	.88	.89	.87	.98	.93	.95	.99	.02	.07	.05	.01
2nd Item	.60	.81	.89	.80	.77	.96	.70	.95	.90	.04	.30	.05	.10
3rd Item	.69	.84	.83	.80	1	.94	.95	.99	.77	.06	.05	.01	.23
**Mean**						**.96**	**.86**	**.96**	**.89**	**.04**	**.14**	**.04**	**.11**
												**Mean**	**.08**

**Table 4:** Reliabilities, consistency and method-specificity coefficients in Study 2.

		Reliability					Consistency				Method-specificity			
	Academic	Math	French	English	Phys Ed	Math	French	English	Phys Ed	Math	French	English	Phys Ed
Intrinsic													
1st Item	.69	.79	.75	.77	.79	.34	.37	.22	.05	.66	.63	.78	.95
2nd Item	.72	.77	.72	.81	.80	.34	.44	.21	.07	.66	.56	.79	.93
3rd Item	.76	.81	.77	.88	.80	.26	.43	.17	.06	.74	.57	.83	.94
**Mean**						**.31**	**.41**	**.20**	**.06**	**.69**	**.59**	**.80**	**.94**
												**Mean**	**.76**
Identified													
1st Item	.61	.81	.64	.72	.63	.26	.32	.21	.26	.74	.68	.79	.74
2nd Item	.59	.77	.68	.71	.50	.28	.38	.22	.10	.72	.62	.78	.90
3rd Item	.76	.81	.71	.74	.71	.31	.41	.30	.15	.69	.59	.70	.85
**Mean**						**.28**	**.37**	**.24**	**.17**	**.72**	**.63**	**.76**	**.83**
												**Mean**	**.74**
Introjected													
1st Item	.59	.66	.85	.80	.58	.88	.87	.84	.64	.12	.13	.16	.36
2nd Item	.50	.79	.56	.86	.67	.62	.93	.56	.48	.38	.07	.44	.52
3rd Item	.56	.74	.61	.70	.57	.85	.98	.77	.50	.15	.02	.23	.50
**Mean**						**.78**	**.93**	**.72**	**.54**	**.22**	**.07**	**.28**	**.46**
												**Mean**	**.26**
External													
1st Item	.51	.76	.92	.72	.61	.85	.64	.69	.71	.15	.36	.31	.29
2nd Item	.64	.65	.80	.65	.77	.90	.95	.79	.50	.10	.05	.21	.50
3rd Item	.55	.79	.78	.71	.63	.76	.97	.80	.64	.24	.03	.20	.36
**Mean**						**.84**	**.85**	**.76**	**.62**	**.16**	**.15**	**.24**	**.38**
												**Mean**	**.23**

**Table 5:** Latent correlations with student’s self-concept in Study 1.

	Mathematics	Science	Writing	Reading	School
Mathematics					
Intrinsic	80***	.23**	-.30**	-.31***	.15
Identified	21*	.10	-.06	-.17	.16
Introjected	-.08	.14	-.25*	-.01	.08
External	-.10	.13	-.11	.23	-.19
Science					
Intrinsic	.19*	.77***	-.20**	-.02	.02
Identified	.06	.60***	-.11	-.05	.10
Introjected	.06	.22*	-.08	-.04	.13
External	.06	.13	-.11	.14	.02
Writing					
Intrinsic	-.19*	.03	.56***	.19*	-.01
Identified	.05	.12	.08	.04	.17
Introjected	.13	.12	-.17	-.08	.04
External	.22	-.03	-.22	.14	-.01
Reading					
Intrinsic	-.25**	.15	.04	.67***	.01
Identified	-.09	.12	-.02	.01	.04
Introjected	.29*	.03	-.07	-.23	.07
External	.09	-.09	-.13	-.06	-.13
School					
Intrinsic	-.03	.22**	.07	.02	.37***
Identified	.03	.17	.08	.17	.20*
Introjected	-.08	.04	.16	.00	-.30***
External	.04	.11	.06	-.11	-.25**

*Note*. All coefficients are standardized correlations.

* p < .05

** p < .01

*** p < .001

**Table 6:** Latent correlations with student’s self-concept in Study 2.

	Mathematics	French	English	Phys Ed	School
Mathematics					
Intrinsic	.74***	-.35**	-.17*	.15*	.07
Identified	.37***	-.19**	-.07	-.07	-.00
Introjected	-.05	.16	.03	-.20	.05
External	-.04	.11	.06	-.07	19*
French					
Intrinsic	-.26***	.60***	-.05	.01	.07
Identified	-.21**	.34***	-.02	.02	-.01
Introjected	-.08	.18	-.09	-.05	-.05
External	.29	-.07	.00	-.03	.23
English					
Intrinsic	-.16*	-.06	.57***	-.10	.04
Identified	-.20**	.01	.41***	-.14*	.13*
Introjected	-.08	.06	.20*	-.17	-.08
External	.19	.07	-.01	.09	.11
PhysEd					
Intrinsic	-.04	-.04	-.07	.75***	.17***
Identified	-.03	-.03	-.08	.59***	.14*
Introjected	.02	-.15	-.10	.30***	.17
External	.11	.04	.07	-.13	.10
School					
Intrinsic	-.06	-.03	.08	-.06	.35***
Identified	-.03	-.06	.12	.10	.33***
Introjected	.03	-.03	.18*	.11	.04
External	.06	-.08	.00	.08	-.10

*Note*. All coefficients are standardized correlations.

* p < .05

** p < .01

*** p < .001

**Table 7:** Latent correlations with external criterion (i.e., students’ teacher-rated achievement) in Study 1.

	Mathematics	Science	Writing	Reading	School
Mathematics					
Intrinsic	.17*	.13	-.07	.02	.10
Identified	-.11	-.04	-.10	-.10	-.09
Introjected	.06	.13	.02	.05	.14
External	-.07	-.05	-.13	-.19	-.16
Science					
Intrinsic	.06	.15	-.06	.05	.01
Identified	-.07	.07	-.11	-.07	-.09
Introjected	.02	.16	-.03	.06	.08
External	.03	-.02	-.08	.02	-.09
Writing					
Intrinsic	-.20**	-.12	-.07	-.09	-.09
Identified	-.05	.08	-.06	-.00	.02
Introjected	-.05	.05	.02	.01	.06
External	.09	.04	.07	.01	.02
Reading					
Intrinsic	.07	.15*	.19*	.25**	.14*
Identified	-.08	-.02	-.00	-.03	-.05
Introjected	-.05	.13	-.03	-.04	.09
External	-.08	-.05	-.08	-.14	-.11
School					
Intrinsic	-.02	.03	.03	-.02	.04
Identified	.03	.12	.06	.01	.12
Introjected	-.36***	-.38***	-.35***	-.42***	-.40***
External	-.28***	-.23**	-.32***	-.30***	-.26**

*Note*. All coefficients are standardized correlations.

* p < .05

** p < .01

*** p < .001

### Testing the specificity hypothesis

#### Reliability, consistency and method-specificity coefficients (Models 1a and 1b)

Our main hypothesis was that the specificity to the school subject level would differ between autonomous motivation and controlled motivation. That is, we were expecting autonomous motivation to be more specific than controlled motivation. Thus, we would expect that the true variance shared at the school subjects level would be greater for autonomous than for controlled motivation and, conversely, that the true variance shared at the contextual level (i.e., the school level) would be smaller for autonomous than for controlled motivation.

This assumption was tested using the CTCM-1 model, in which the strength of the relationships between the contextual trait and the observed variables and between the school-subject- specific latent constructs and the observed variables were evaluated with consistency and method-specificity coefficients. The ***consistency coefficient*** indicates the proportion of true variance that is shared at the school level. The ***method-specificity*** coefficient represents the proportion of true variance of the items that is shared at the school subjects level (see Eid et al., 2008, for more details on these coefficients). Table 3 and Table 4 present the reliability, consistency and method-specificity coefficients for the CTCM-1 models in Study 1 and Study 2 respectively. The ***reliability coefficient*** represents the proportion of the total variance that is not due to measurement error. The reliabilities coefficients for all the items in both studies are globally satisfactory.

First, in Study 1 (Table 3), the consistency coefficients for intrinsic motivation (means .30–.48) and identified regulations (means .22–.62) were lower than for introjected (means .73–.90) and external regulations (means .86–.96). Consequently, the method-specificity coefficients were higher for intrinsic motivation (means .52–70) and identified regulation (means .38–.53) than for introjected (means .10-.18) and external (means .04-.11) regulations. The results show the same pattern in Study 2 (Table 4). Taken together, these coefficients indicate that school-subject-specific deviations from the academic trait were more strongly related to observed variables for intrinsic motivation and identified regulation than for introjected and external regulations. True item variance is explained mostly at the situational level for autonomous motivations (from 38% to 70% in Study 1, and from 59% to 94% in Study 2) and at the contextual level for controlled motivations (from 73% to 96% in Study 1, and from 54% to 93% in Study 2).

#### Latent correlations between autonomous and controlled motivations and self-concepts (Models 2a and 2b)

Table 5 and Table 6 present the results of the standardized latent correlations between autonomous and controlled academic motivations and self-concepts for Study 1 and Study 2 respectively. All correlations between specific self-concepts and matching intrinsic motivation were high, positive, and statistically significant in Study 1 (.80, .77, .56, and .67 for mathematics, science, writing, and reading, respectively) as well as in Study 2 (.74, .60, .57 and .75 for mathematics, French, English and physical education, respectively). More importantly, these correlations were higher than those connecting intrinsic motivation for a given subject to a non-matching self-concept measure (i.e., correlation connecting intrinsic motivation for math to science self-concept). Only two significant correlations were found between self-concepts and identified regulations in Study 1 (.21 and .60 for mathematics and science, respectively) whereas the four correlations were significant in Study 2 (.37, .34, .41 and .59 for mathematics, French, English and physical education, respectively). One positive significant correlation was found between science self-concept and introjected regulation in Study 1 (.22 for science) and two in Study 2 (.20 and .30 for English and physical education, respectively).

Other significant correlations are noteworthy in both studies. For example, self-concept in mathematics was negatively correlated to intrinsic motivation in writing and reading (-.19 and-.25, respectively) in Study 1and to intrinsic motivation in French and English (-.25 and-.16, respectively) but also to identified regulation in French and English (-.26 and-.16, respectively) in Study 2. Self-concept in mathematics was also positively correlated to intrinsic motivation in science in Study 1 (.19). Similarly, self-concept in writing was negatively correlated to intrinsic motivation in mathematics and science (-.30 and-.20, respectively), self-concept in reading was negatively correlated to intrinsic motivation in mathematics (-.31) but positively to intrinsic motivation in writing (.19), and self-concept in science was positively correlated to intrinsic motivation in mathematics (.23) in Study 1. In Study 2, intrinsic motivation in mathematics was negatively correlated to self-concept in French and English (-.35 and-.17, respectively) and positively correlated to self-concept in physical education (.15). Moreover, in Study 2, other significant correlations appeared between self-concept and non-corresponding identified regulation. More specifically, self-concept in mathematics was negatively correlated to identified regulation in French and in English (-.21 and-.20 respectively) whereas self-concept in French was negatively correlated to identified regulation in mathematics (-.19). Thus, there are positive correlations between intrinsic motivation in a given school subject and non-corresponding self-concept when the subjects from similar academic domains (e.g., the mathematical domain could explain the positive correlation between science intrinsic motivation and mathematics self-concept), but negative ones when on the subjects are from different ones (e.g., verbal vs. mathematics; the negative correlation between writing intrinsic motivation and mathematics self-concept) in both studies. In Study 2, we also found three negative correlations with non-corresponding self-concept for identified regulations. These results corroborate the idea that positive correlations connecting intrinsic motivation to non-corresponding self-concept dimensions depend on whether the domain is verbal or mathematical [31].

Note also that at the academic level, the pattern of correlations between academic self-concept and regulation types supports the specificity hypothesis in both studies. First, regulation types for the general academic dimension were significantly correlated to general academic self-concept in Study 1 (.37 and .20 for intrinsic motivation and identified regulation, and-.30 and-.25 for introjected and external regulations) and in Study 2 (.35 for intrinsic motivation and .33 for identified regulation), in line with SDT predictions. Moreover, regulations in each school subject were not associated with general academic self-concept in Study 1.

#### Latent correlations between autonomous and controlled academic motivations and achievement (Model 3)

Table 7 presents the results of the standardized latent correlations between autonomous and controlled academic motivations and students’ teacher-rated achievement rated by teacher. Correlations between specific students’ achievement and matching intrinsic motivation were positive and statistically significant for mathematics and reading (.17, and .25 respectively), but nearly significant for science (.15, p < .05). Four other significant correlations between students’ achievement and intrinsic motivation were found: Intrinsic motivation in writing was negatively correlated to students’ achievement in mathematics (-.20) and intrinsic motivation in reading was positively correlated to students’ achievement in science, writing and school (.15, .19, and .14, respectively). These results are thus similar to those observed with self-concept dimensions.

None of the correlations between identified, introjected or external regulations and students’ teacher-rated achievement were statistically significant at school-subject-specific level. However, the correlations between controlled motivations at the academic level were negatively related to students’ teacher-rated achievement in all school subjects. More specifically, it appears that controlled motivations are not school-subject-specific but predict achievement negatively in each specific school subject. In other words, when we measure controlled motivation in specific school subjects, we are not measuring something specific to a school subject, but rather something more global or contextual.

One way to test the previous finding more stringently is to compare the obtained CTCM-1 solution to a classical CFA one where all indicators are caused solely by their respective latent constructs. More specifically, if the classical CFA solution reveals that introjected and external school subject regulations are negatively related to achievement in school subjects, this would mean that the CTCM-1 solution captures a large portion of variance that is contextual and not school-subject-specific. Correlations from the classical CFA solution are in line with this idea. More specifically, in a classical CFA model, all twenty correlations between controlled motivations and achievements were found to be negative and significant for introjected (between-.14 and-.30) and external (between-.16 and-.29) motivations. Therefore, these results demonstrate that the common variance shared at the specific and contextual levels attenuates the correlations between school subject regulations and students’ achievement at the situational level. This supplementary analysis thus provides relatively good support for the idea that controlled motivation is not specific but rather contextual at this age.

## Discussion

The aim of this study was to test the specificity of autonomous and controlled motivations in the academic context. A multidimensional and hierarchical structure had been previously proposed by Vallerand [5]. However, no study to date had tested a model combining relationships among the regulations at the school subject level and at various hierarchical levels of generality. Our findings based on variance components and various correlations between latent constructs (i.e., correlations connecting autonomous and controlled motivations to self-concept and achievement) suggest that some regulation types may be more school-subject-specific depending on their level of self-determination. That is, the results from Models 1a and 1b (variance components) as well as those from Models 2a and 2b (correlations connecting regulation types with self-concepts) and from Model 3 (correlations connecting regulation types with performance) corroborate this hypothesis.

### The multidimensional aspect of autonomous and controlled motivations

Few studies have considered the multidimensional aspect of academic motivation using a between-level-of-generality or multiple-school-subject approach in addition to the traditional one-dimensional approach. However, other research fields (e.g., academic self-concept) [21] have demonstrated the relevance of investigating multidimensionality across school subjects. Demonstrations of the relationships between achievement and self-concepts across school subjects [32] have led to further investigations of the structure of academic self-concept to better understand the underlying processes. Regarding autonomous and controlled academic motivations, a previous study by Guay et al. [14] highlighted that autonomous and controlled motivations do not differentiate the same way across school subjects. In the present study, we demonstrated that autonomous motivation could be more differentiated because it is more specific to school subjects, whereas this was less the case for controlled motivation.

Intrinsic and identified motivations for each school subject were positively related to specific self-concepts. Moreover, associations were found between intrinsic motivation in various school subjects (e.g., a negative relationship between intrinsic motivation in math and intrinsic motivation in reading in Study 1, or a negative relationship between intrinsic motivation in math and intrinsic motivation in French in Study 2) and between intrinsic motivation and identified regulation in a given school subject and self-concept in another (e.g., between intrinsic motivation in math and all other school subject self-concepts in both studies, between identified regulation in math and French and non-corresponding self-concepts in Study 2). These results regarding the multidimensionality and specificity of autonomous motivation point to the need to investigate the relationships between autonomous and controlled motivations, self-concept, and achievement in one or multiple school subjects. The correlations found between intrinsic motivation in mathematics and other school subject self-concepts (e.g., positive correlation with science and negative correlations with writing and reading in Study 1, positive correlation with physical education and negative correlations with French and English in Study 2) reproduced previously demonstrated negative relationships between verbal and mathematical domains in self-concept studies [32]. Therefore, future studies should integrate autonomous motivation in multiple school subjects to examine how students develop their self-concept and academic achievement over time. Even if the reciprocal causal ordering between self-concept and achievement is now well-established in the literature [33], the motivational mechanisms underlying these effects have not been particularly studied. More specifically, in light of our results, we would expect the relationship between self-concept and achievement in a given school subject to be influenced not only by autonomous motivation for that school subject but also for other school subjects.

A few studies have explored the relationships between autonomous and controlled academic motivations, self-concept, and achievement [20, 34, 35]. These studies were conducted at the school level, using cross-sectional or longitudinal designs to test mediation models or additive models between these constructs [14]. The results of these studies support a mediational model of motivation (academic self-concept predicts motivation, which in turn predicts academic achievement). However, this mediational model has been supported at the school level only, using a relative autonomy index that disregards the potential differences in specificity differentiation effects found in our studies. Therefore, little is known about the situational level while autonomous and controlled motivations are considered, and we suggest that more complex relationships across subjects could emerge.

Our results therefore open up new research avenues concerning the relationships among constructs in various school subjects by proposing and testing a subject-specific model with a multidimensional component.

### The hierarchical aspect of autonomous and controlled academic motivations

The HMIEM proposed by Vallerand [5] postulates a school-subject differentiation of motivation at various levels of generality. This model has received some support from empirical studies having tested the relationships between motivation at various levels of generality as well as the specificity of each level by relating antecedents and outcomes to motivation at the situational and contextual levels. However, we believe that the results of our study raise some questions about the HMIEM. Indeed, we showed that the specificity of the within-school-subject motivational constructs depends on the level of self-determination. Compared to autonomous motivation, controlled motivation reported by students in a given school subject appears to be more related to a global trait (i.e., motivation at the academic level). Studies on the HMIEM have not yet considered this possibility. The relative autonomy index was developed and applied without considering the fact that the specificity of the regulations could differ depending on the level of self-determination.

Several implications may be derived from these findings. First, they have implications for the relative autonomy index (RAI). The RAI combines various types of motivations under a single construct [6]. More specifically, the RAI formula is based on the level of self-determination associated with each regulation. For example, a weight of +2 is assigned to intrinsic motivation, +1 to identified regulation, -1 to introjected regulation, and -2 to external regulation. The RAI thus contrasts the relative amount of autonomous motivation to the one of controlled motivation and offers the possibility of conducting more parsimonious analyses. A positive score on the RAI means that individuals endorse more autonomous motives than controlled ones. Because controlled motivations were found to be less specific at the school subject level, the RAI variations at different levels of generality would be due mostly to autonomous motivation variations at these levels and could not be attributable to controlled motivation variations. Therefore, the specificity of the various levels obtained in HMIEM studies could simply be an artifact of the specificity of autonomous motivation supported by our results.

Broadly speaking, we believe that our results bring into question the operationalization of autonomous and controlled motivations when multiple domains or multiple levels of generality are considered. Different psychological processes could be involved for motivational types that are more contextual (introjected and external regulations) than specific (intrinsic and identified regulations). In fact, autonomous motivation assessed in a specific school subject has been described as more specific, and therefore less related to the contextual level, compared to controlled motivation assessed in the same school subject. We believe that the distinction between autonomous and controlled motivations should be accounted for when investigating multiple domains or multiple levels of specificity and, accordingly, composite scores, such as the relative autonomy index, should not be used in these studies.

### Theoretical issues concerning the hierarchical differentiation effect for autonomous and controlled motivations

In past studies [16, 17, 18], autonomous and controlled motivations have both been conceptualized to fluctuate from one specific domain to another without considering that regulation types could be more or less specific. Our results show that such a distinction has theoretical implications for research in the school domain. Perhaps one of the most important consequences of these findings resides in our understanding of the mechanisms involved in the development of regulation types in school subjects. Two main types of antecedents of motivational resources are commonly postulated and tested in studies: social environments and global personality orientations [36]. These antecedents have also been considered to influence autonomous and controlled motivations similarly according to specific school subjects without considering hierarchical differentiation effects. However, as controlled motivation has been demonstrated to be less specific than autonomous motivation, we could infer that global personality orientations would be more responsible for students’ controlled motivation in a specific school subject than for their autonomous motivation. We could also infer that autonomy support provided by teachers in a specific school subject would impact more autonomous motivation (i.e., because they are more school-subject-specific) and less controlled motivation. Our study thus challenges the traditional view of motivational antecedents and students’ motivation in a specific school subject. The differentiation effect tested here offers a new perspective on autonomous and controlled motivations in a specific school subject by showing that controlled motivation could be more contextual construct. Therefore, we believe that special attention must be paid to the contextual-school-subject distinction of autonomous and controlled motivations in future research. We further believe that it is important to reconsider past results in light of these findings.

### Limitations and directions for future research

These studies include certain limitations that affect the results interpretation. First, the sample size is small in both studies. The complexity of the models and the number of estimated parameters would require larger samples, especially for variance estimates. However, results concerning the parameters of interest in our manuscript (i.e., factor loadings, correlations between regulations and self-concept and achievement) were similar in less complex models that considered only one regulation at the time. Second, these studies are the firsts to test a model with this degree of complexity. The results should be considered with caution, especially with regard to their magnitude. The school subjects considered and the measurement scales may have influenced the level of the differences in specificity effects. Third, we found rather low CFI and TLI values in our global model. These weaknesses are not problematic and could simply reflect the fact that introjected and external motivations are less likely to be modelled with a hierarchical organization of constructs having a general construct at the apex and subject-specific constructs separated across domains. Fourth, it may have been preferable to use an objective measure of each student’s achievement that is directly comparable across various groups or classes. However, research has shown that marks given by elementary teachers correlate with those given by other teachers having taught the same students in a different school year, thereby providing some support for the reliability of this kind of measure (see [37] for a test–retest correlation of .69). Fifth, the design used is cross-sectional. It is therefore difficult to conclude any causal relations between motivation types, self-concepts, and achievement scores. Moreover, the students were all from Switzerland with French as their mother tongue. It is thus important to replicate these results in various student populations from different countries and cultural backgrounds.

In sum, researchers have pointed out the need to investigate autonomous and controlled motivations in school from a more multidimensional, structured perspective. Our findings make a substantial contribution in that they demonstrate that some motivation types proposed by SDT are more school-subject-specific than others. This could have important practical implications by helping in the development of more targeted motivational interventions.
